# Deficiency in Nucleotide Excision Repair Family Gene Activity, Especially ERCC3, Is Associated with Non-Pigmented Hair Fiber Growth

**DOI:** 10.1371/journal.pone.0034185

**Published:** 2012-05-16

**Authors:** Mei Yu, Robert H. Bell, Maggie M. Ho, Gigi Leung, Anne Haegert, Nicholas Carr, Jerry Shapiro, Kevin J. McElwee

**Affiliations:** 1 Department of Dermatology and Skin Science, University of British Columbia, Vancouver, Canada; 2 Vancouver Coastal Health Research Institute, Vancouver, Canada; 3 Prostate Centre, Vancouver General Hospital, Vancouver, Canada; 4 Department of Surgery, University of British Columbia, Vancouver, Canada; Louisiana State University and A & M College, United States of America

## Abstract

We conducted a microarray study to discover gene expression patterns associated with a lack of melanogenesis in non-pigmented hair follicles (HF) by microarray. Pigmented and non-pigmented HFs were collected and micro-dissected into the hair bulb (HB) and the upper hair sheaths (HS) including the bulge region. In comparison to pigmented HS and HBs, nucleotide excision repair (NER) family genes *ERCC1*, *ERCC2*, *ERCC3*, *ERCC4*, *ERCC5*, *ERCC6*, *XPA*, *NTPBP*, *HCNP*, *DDB2* and *POLH* exhibited statistically significantly lower expression in non- pigmented HS and HBs. Quantitative PCR verified microarray data and identified ERCC3 as highly differentially expressed. Immunohistochemistry confirmed ERCC3 expression in HF melanocytes. A reduction in ERCC3 by siRNA interference in human melanocytes *in vitro* reduced their tyrosinase production ability. Our results suggest that loss of NER gene function is associated with a loss of melanin production capacity. This may be due to reduced gene transcription and/or reduced DNA repair in melanocytes which may eventually lead to cell death. These results provide novel information with regard to melanogenesis and its regulation.

## Introduction

Hair follicles produce hair fiber during regular cycles of active growth and relative quiescence [1]. Hair follicle (HF) cycling consists of an active, hair fiber producing, growth phase called anagen; catagen, a transition phase involving regression of the hair follicle; and telogen as a resting phase. Hair color is provided by pigments produced by melanocytes and the behavior of HF resident melanocytes is tightly coupled to the hair growth cycle [2], [3].

HF melanocyte stem cells (MSC) are localized in the outer root sheath (ORS) near the HF “bulge” region, where HF epithelial stem cells reside [4]. The MSCs are maintained in this niche environment throughout the entire hair cycle [5]. When a new anagen growth phase begins, epithelial stem cells and MSCs undergo a brief period of proliferation via coordinated signaling involving Wnt expression in both cell populations [6]. The epithelial progenitor cells regenerate the anagen follicle structure including the hair bulb [7]; while the melanocyte progenitors home to the HB as it forms and differentiate into mature melanocytes [5], [8]. The interactions between mature melanocytes in the HB and the surrounding hair matrix keratinocytes and dermal papilla cells coordinate melanogenesis [9], [10]. Hair becomes pigmented due to the transfer of melanin from HB melanocytes into the proliferating hair matrix keratinocytes. HB melanocytes are terminally differentiated and undergo apoptosis when the hair follicle involutes in early catagen [11].

Numerous factors have been recognized that may affect hair pigmentation, including general metabolism and nutritional status, hair-cycle dependent changes, body distribution, racial and gender differences, hormone-responsiveness, genetic defects and age-associated changes [12]. As with any multi-step process, there are many positive and negative regulators and pathways controlling hair pigmentation including growth factors, cytokines, hormones, neuropeptides and neuro-transmitters, eicosanoids, and cyclic nucleotides [13]. These inputs into melanogenesis regulation have made understanding the molecular pathways and identifying key factors in determining MSC and mature melanocyte activity, and the development of white hair, difficult.

Recent advances in molecular genetics offer some intriguing clues to explain the basis of the diversity in hair pigmentation. Currently, the ‘free radical’ theory is the most popular explanation for the development of non-pigmented hair growth development with age [14]. Studies on epidermal melanocyte aging suggest that reactive oxygen species-mediated damage to nuclear and mitochondrial DNA leads to mutation accumulation in melanocytes [10], [12]. Parallel dysregulation of anti-oxidant mechanisms or pro/anti-apoptotic factors is also likely to occur within the cells [12]. Due to irreparable DNA damage, MSCs may eventually differentiate into ectopically pigmented mature melanocytes (EPMs), without renewing themselves in the hair bulge niche [15], [16]. These EPMs are subsequently eliminated in late anagen. Impaired self-renewal of MSCs through these processes may result in progressive “hair graying”, as more hair follicles produce non-pigmented white hair in subsequent hair growth cycles [15].

In this study, we used microarrays to determine differences in gene replicate variance between pigmented and non-pigmented HFs. We identified the nucleotide excision repair (NER) pathway may play an important role in modulating MSC attrition and the development of non-pigmented hair growth. Further, we found a key factor of the NER pathway, ERCC3, which may play a key role in maintaining and regulating the fate and behavior of MSCs and mature melanocytes.

## Results

### Analysis of Gene Expression Differences

Gene expression of the tissue groups indicated that 388 genes were differentially expressed between pigmented hair sheaths (HS) and non-pigmented HS with statistical significance (Table S2). 2289 genes were differentially expressed between pigmented HBs and non-pigmented HBs (Table S1). Among the differential expression gene lists, only a few genes were up-regulated in non-pigmented, white HFs, most genes were down regulated as compared to pigmented HFs.

### Differences in Gene Ontology Categorization

We functionally annotated Table S1 and Table S2 using Gene Ontology (GO) terms [7]. The statistically significant GO term results (adjusted P<0.05) for genes significantly differentially expressed in pigmented and non-pigmented HS and pigmented and non-pigmented HBs are shown (Table S3and Table S4). The GO analysis revealed that differentially expressed genes between pigmented and non-pigmented HBs (Table S1) were significantly enriched in some dominant functional categories, such as “angiogenesis” (24 genes), “regulation of cell death” (113 genes) and “positive regulation of cell death” (60 genes). These categories may relate to the dysfunction of melanocytes.We also found gene enrichment of some categories under the blanket term of “metabolic process”, including “melanin metabolic process” (5 genes) and “pigment biosynthetic process” (9 genes) which may relate to the reduction of melanin product (Table S3).

Several gene function categories were identified with enrichment in significantly differentially expressed genes between pigmented HS and non-pigmented HS (Table S1), such as “secretion” (15 genes), “regulation of cell communication” (45 genes) and “signal transduction” (90 genes) under the primary category of “biological regulation”. Further, “regulation of cellular response to stress” (8 genes) and “regulation of response to stress” (13 genes) under the category of “response to stimulus” were also identified (Table S4). In addition, gene enrichment of the subcategory “response to radiation” (13 genes), which is under the term “response to stimulus”, was also observed (Table S3).

### Pathway Analysis

Genes in Table S1 and Table S2 were classified and grouped into different pathways imported from the Kyoto encyclopedia of Genes and Genomes (KEGG) [17]. The “response to radiation” pathway, which includes genes involved in DNA repair, was identified with significant difference between pigmented HB and HS and non-pigmented HB and HS.

### Gene Expression Validation

**Figure 1:**
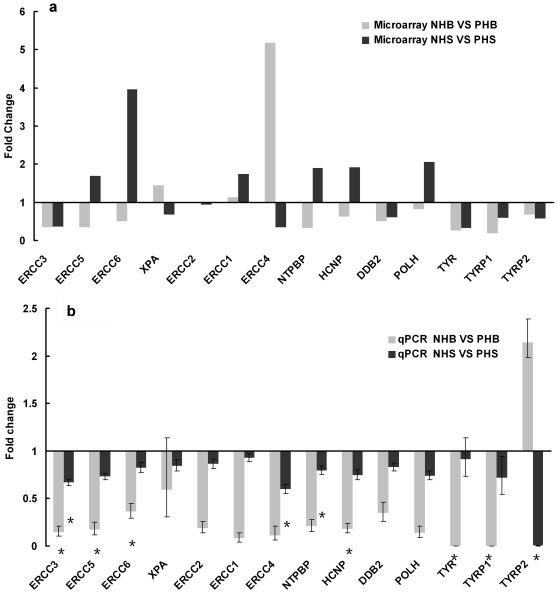
Quantitative PCR analysis of selected NER genes. Quantitative expression of 11 genes identified from microarray data, with known functional significance related to NER, was determined (a). We also reconfirmed differential gene expression of melanocyte markers (tyrosinase, tyrosinase related protein 1) as validation (b). Trends for upregulation or down-regulation were largely consistent with microarray data.

From the “response to radiation” pathway, we selected 11 genes with known functional significance associated with NER for further evaluation by quantitative polymerase chain reaction (qPCR). We also reconfirmed differential gene expression of melanocyte markers, tyrosinase, tyrosinase related protein 1 and tyrosinase related protein 2, to validate our study. The trends, whether for increased or decreased gene expression, were largely, though not entirely, consistent with the microarray data (Fig. 1).

Because the sensitivity of the qPCR assay is usually higher than that of the microarray assay, discordant results for individual genes could be due to differences in detection limits for the two assays [18]. Poorer correlations between qPCR and microarray assay data were found for genes that exhibited small fold-change differences (<1.5 fold) such as for ERCC5 and ERCC1. Another possible explanation is that different transcript sequences may be recognized by microarray probe sets versus the qPCR primers [19]. The targeting of different sequences might explain the discrepancies found in relative gene expression (>1.5 fold), such as for ERCC4 and TYRP2.

The ERCC3 gene mRNA showed statistically significant downregulation in both non-pigmented HB (0.144 fold) and HS (0.66 fold) by qPCR. This gene, which showed the most consistency in expression trends between the two assays, became a particular focus of interest for further investigation.

### Expression of ERCC3 by Immunohistology

**Figure 2:**
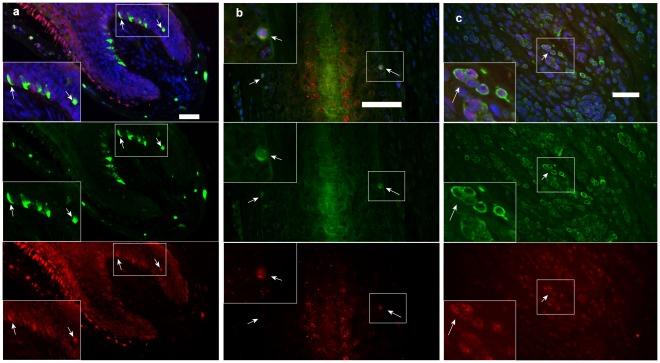
Co-expression of ERCC3 and melanocyte marker gp100 in hair follicles and pigmented nevi. Dual label immunofluorescence defined ERCC3 expression in relation to mature melanocytes and melanocyte stem cells in terminal hair follicles in normal scalp biopsies and nevi tissues. A few dual labeled gp100 positive (green) mature melanocytes and ERCC3 positive (red) cells were detected in pigmented hair follicle bulb regions (a). Dual labeling of isolated cells was also located close to the hair bulge region, suggesting these may be ERCC3 positive melanocyte stem cells (b). Moderate ERCC3 protein expression was detected in mature melanocytes with strong gp100 expression in nevi tissues (c). Bar = 40 µm.

Dual immunofluorescence labeling was conducted for ERCC3 expression in relation to gp100, a marker for mature melanocytes and premature melanocytes, in terminal scalp HF and nevi tissues. In normal scalp, dual labeled mature melanocyte and ERCC3 positive cells were readily detectable in HF bulb region (Fig. 2a). Dual labeling of isolated cells, with no visible pigmentation, was also located close to the hair bulge region, suggesting these may be ERCC3 positive melanocyte progenitors (Fig. 2b). ERCC3 expression was also detected in mature melanocytes with strong gp100 expression in highly pigmented nevi tissues (Fig. 2c).

### Melanogenic Enzyme mRNA Levels after Transfection with ERCC3 siRNA

**Figure 3:**
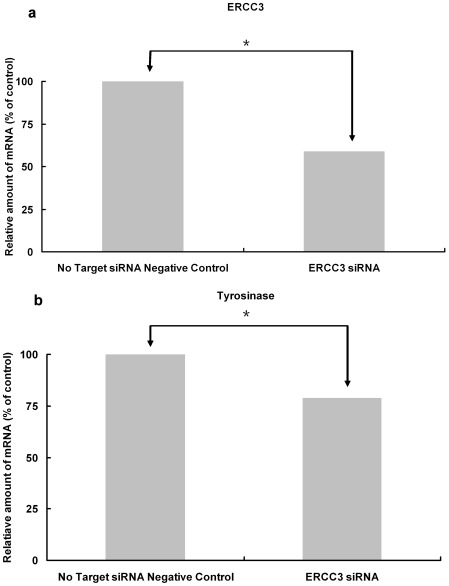
Effects of ERCC3 siRNA transfection on ERCC3 and TYR mRNA levels in human epidermal melanocyte cells. Cells transfected with ERCC3 siRNA displayed a statistically significantly lower level of ERCC3 mRNA than negative control siRNA transfected melanocytes (a). There was a corresponding reduction in TYR mRNA levels in ERCC3 siRNA transfected cells compared with negative control siRNA transfected cells (b). These experiments were repeated 3× with similar results. *, *p*≤0.05 *versus* control.

To examine whether ERCC3 siRNA could selectively knockdown ERCC3 gene expression, we conducted qPCR on transfected melanocytes (Fig. 3). Cells transfected with ERCC3 siRNA displayed a statistically significantly lower level of ERCC3 (59% reduced) mRNA compared to negative control siRNA transfected melanocytes. Melanin synthesis is regulated by tyrosinase (gene; TYR). We also detected a reduction in TYR mRNA levels in ERCC3 siRNA transfected cells (79% reduction) compared with negative control siRNA transfected cells, indicating that a deficiency in ERCC3 is associated with a relative reduction in melanogenesis activity.

### Tyrosinase Activity in ERCC3 Transfected Cells

**Figure 4:**
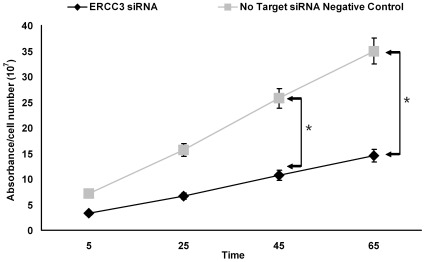
Effect of ERCC3 siRNA transfection on tyrosinase activity in ERCC3 siRNA and negative control siRNA transfected human epidermal melanocytes. Cells were transfected with ERCC3 siRNA and negative control siRNA tyrosinase activity were evaluated by measuring l-DOPA oxidation at several different time points at 475 nm. The data were normalized to the cell number. The data show the means ± S.D. of three experiments performed in duplicate. *, *p*≤0.05 *versus* control.

Tyrosinase is a rate-limiting enzyme in melanin synthesis. To investigate the effect of ERCC3 on pigmentation, we detected the activity of tyrosinase [13], [20]. To determine if there was a difference in tyrosinase product activity between ERCC3 siRNA and negative control siRNA transfected melanocytes, the amount of DOPA chrome formed was measured (Fig. 4) [21]. Compared with no target siRNA transfected cells, tyrosinase activity was statistically significantly reduced in ERCC3 siRNA transfected cells at 40 and 60 minutes. This result indicates ERCC3 deficiency results in a relative lack of tyrosinase activity consistent with reduced melanin production. The cell numbers did not show significant difference between ERCC3 transfected cells and negative control siRNA transfected cells (not shown).

## Discussion

### Global Changes in Gene Expression in White Hair Follicles

We compared the global gene expression signature of pigmented and non-pigmented HS and HB by microarray with the aim of detecting differently expressed genes related to pigmentation regulation and white hair. Unsupervised SAM revealed a high degree of similarity in gene expression confirming the homogeneity of the common tissue source for the pigmented and non-pigmented HSs and HBs. Low gene expression variation suggested the heterogeneity of pigmented and non-pigmented HFs is low. Nevertheless, we found some genes with differential expression between pigmented and non-pigmented HF parts. Compared with pigmented HF gene expression profiles, a small group of genes were identified as significantly over-expressed in non-pigmented HFs; on the other hand, a much larger number of significantly differentially expressed genes were identified with a tendency towards decreased expression in non-pigmented HFs compared with pigmented HF gene expression profiles. These results imply that increased dysfunction is the outcome of most molecular processes associated with white HFs.

White hair is not simply due to a loss of melanocytes, rather melanocyte function becomes progressively disordered in non-pigmented HFs. Normally amelanotic MSCs in the outer root sheath differentiate and may begin to produce pigment [22]. In contrast, melanocytes in the HB become amelanotic, but continue to survive [23]. The amelanotic melanocytes of white HF appear to be recruitable for repigmention if given the necessary stimulation [24], [25]. The hair “aging” process may be first dominated by the melanocyte “aging” process at the gene expression level.

### Gene Expression in White Hair Follicle Bulbs

GO analysis of the differentially expressed genes between pigmented and non-pigmented HBs indicated there were significant changes in specific functional gene groups. We found negative regulation of genes in the categories of “melanin metabolic process” (5 genes) and “pigment biosynthetic process” (9 genes) in white HF bulbs consistent with a loss of melanin production in mature melanocytes. We also found significant gene expression changes in the categories of “regulation of cell death” (109 genes) and “positive regulation of cell death” (58 genes) consistent with dysfunctional apoptosis in non-pigmented HBs. This may be more likely to occur in mature melanocytes because HB keratinocytes and fibroblasts reportedly have higher antioxidant enzyme activities than melanocytes [3], [14].

Incidence of HB melanocyte apoptosis in the aging human HF may be an indicator of oxidative stress induced tissue damage and associated mitochondrial DNA damage. Melanin-synthesis generates H_2_O_2_ and other free radicals via the hydroxylation of tyrosine and the oxidation of DOPA to melanin, placing HF melanocytes under high oxidative stress [14]. Studies show that H_2_O_2_, even in low doses, can induce cell senescence [3]. If not adequately removed due to insufficient oxidative phosphorylation, an accumulation of these reactive oxygen species will generate significant oxidative stress in melanocytes [14]. In our white hair gene expression pattern, if the ontological analyses is performed at biological process category level 4, which classifies genes in categories with more detail than level 3, 20 “oxidative phosphorylation” genes (e.g., ATP synthases and NADH dehydrogenases) were down-regulated in non-pigmented HBs compared with pigmented HBs (data not shown).

Our results suggest non-pigmented hair could be associated with decreased oxidative phosphorylation coupling efficiency and increased superoxide production in HB melanocytes [3], [14]. Melanocytes may be more sensitive to damage than the surrounding cells as unpigmented HFs retain their hair fiber growth capacity. Melanocyte senescence may be a loss of balance between radical generation and anti-oxidative stress and anti-oxidative stress function is likely important in melanocytes for preventing a loss of hair pigmentation.

### Gene Expression in White Hair Follicle Root Sheaths

The outstanding categories, “response to radiation”, “regulation of cellular response to stress” and “regulation of response to stress” were identified with significantly different expression between pigmented HS and non-pigmented HS. These categories include genes which are involved in cell maintenance, gene transcription and DNA repair. Our result is consistent with other studies that suggest radiation-induced stem cell damage can result in non-pigmented hair [15]. While radiation may or may not be directly involved in promoting white hair, activation of melanocyte cells’ protective mechanisms is likely to be similar in the “real world”. Reactive oxygen species (ROS) induced DNA damage (nuclear and mitochondrial) leads, not only to the accumulation of mutations, but also to anti-oxidant mechanisms. The DNA damage must be repaired in order to maintain the genome integrity, growth and productivity. Organisms have evolved unique cellular defense response mechanisms to cope with DNA damage [26]. Within these categories, we found several common genes that belonged to the NER signaling pathway which is a central player in the coordination of gene transcription, DNA repair, damage tolerance, and genome stability. This result suggests DNA damage repair mechanisms in MSCs may play a dominant role in the process of pigmentation loss in hair.

### The DNA Damage Repair Signaling Pathway Modulates the Process of Pigmentation Loss in Hair

Mature melanocytes and MSCs are under different oxidative stress conditions, but the same result may ensue; cellular DNA damage, gene transcription defects, and subsequent progressive dysfunction. DNA injury may compromise gene transcription and cell replication and eventually cause cellular senescence (growth arrest) or cell death (apoptosis) [27]. Based on our results, DNA-repair mechanisms appear to be the common theme in the maintenance of melanocytes and hair pigmentation. NER is a complex process, which removes helix-distorting damage including major UV-induced lesions, bulky chemical adducts, and some forms of oxidative damage [28]. Defects in genome maintenance by NER can induce stem-cell attrition, which is associated with accelerated human aging [29]. NER gene mutations are associated with heritable disorders exhibiting accelerated aging including xeroderma pigmentosum (XP), Cockayne syndrome (CS), and progeria, as well as trichothiodystrophy (TTD) [30]. We found most NER genes were significantly downregulated in white HFs consistent with a reduction of melanocyte activity.

### ERCC3 May be Involved in Modulating the Process of Pigmentation Loss in Hair

Our results showed ERCC3 was statistically significantly down-regulated in white HF. ERCC3 along with ERCC2 are components of the transcription factor IIH (TFIIH) which is involved in gene transcription initiation. The TFIIH complex also plays a key role in NER by opening damaged duplex DNA at the damage site through the action of its two helicases, ERCC2 and ERCC3. Mutation in either ERCC3 or ERCC2 can cause instability of the whole TFIIH complex [31]. A TTD mouse model, with a single Ercc2 gene mutation, prematurely shows an age-related phenotype, including patchy depigmentation and white hair [32]. “Severe” genocopy alterations of Ercc3 cause embryonic lethality in mice, but a “mild” splice site mutation in Ercc3, leading to a 40% reduction of TFIIH levels, enables survival [33]. The cells of the mutant mice demonstrate hypersensitivity to UV radiation and acute oxidative stress. The impact of reduced ERCC3 activity might range from “mild” symptoms, potentially including white hair growth with age, to more severe complications depending on the impact on TFIIH levels.

Insufficient ERCC3 function reduces DNA repair which correlates with an increased cellular sensitivity to oxidative DNA damage, cell death, and consequent depletion of cell renewal capacity similar to ERCC2 deficiency [32]. Moderate deficiency of ERCC3 function may also result in limited unrepaired DNA damage that compromises gene transcription, leading to effective gene inactivation, but without cell death. On the other hand, Gilchrest et al’s study suggested that UV irradiation induces a second UV-protective response, the repair of UV-damaged DNA. Enhanced repair capacity can improve cells’ survival and colony –forming abilities by the up-regulation of a set of genes involved in DNA repair, such as ERCC3 and GADD45, and cell cycle inhibition genes [34]. We did not observe significant cell death in ERCC3 siRNA transfected cells in the short time duration of our functional study. This result suggests reduced ERCC3 function cannot cause immediate melanocyte death, but the previously published studies described suggest it might cause cell senescence and potentially reduce the life-span of melanocytes in the longer term.

Insufficient ERCC3 function may directly reduce the ability to transcribe genes [35] as well as impair transcription-coupled repair [36]. Absence of ERCC3 enhances the transcriptional insufficiency thought to be responsible for several cutaneous abnormalities [32], [37]. Patients with TTD and CS exhibit manifestations that involve transcription deficiency as well as DNA repair defects [38]. We observed a positive correlation between reduced levels of ERCC3 and reduced tyrosinase activity in melanocytes. This result indicates that a relative deficiency of ERCC3 function in melanocytes may slow down transcription efficiency which in turn may affect tyrosinase synthesis and consequently melanin pigment production. Losing hair color is an “aging dependent” process that may be looked as a struggle between ERCC3 related transcription efficiency and progressive oxidative DNA damage.

In conclusion, our data suggest that primary transcription decay is likely related to the process of pigmentation loss in hair. An imbalance between DNA damage and DNA repair transcription capacity may accelerate physiological “hair graying”. We suggest the “aging” of melanocytes due to oxidative stress sensitivity affects hair pigmentation. We propose that reduced ERCC3 activity in HF melanocytes may also reduce transcriptional sufficiency leading to reduced melanogenesis activity. Stochastic damage may also accumulate in MSCs and mature HF melanocytes because of insufficient NER gene function. In the longer term, insufficient NER function may cause cell senescence and death in MSCs and mature melanocytes might also have a reduced life span. Strengthening DNA repair transcription and tapping into this NER pathway, especially ERCC3 may slow down the process of pigmentation loss in hair.

## Materials and Methods

### Hair Follicles and Clinical Information

Human HFs were collected from 4 mm scalp biopsies of healthy individuals who presented with a combination of pigmented and non-pigmented white hair (colloquially described as “salt and pepper” hair). Using a common source for the comparison groups negated any potential differences in gene expression due to differences between individuals in terms of environmental exposure or systemic hormonal activity. The samples were derived from 7 females 43–55 years of age (mean of 49.8 years±SD 4.56 years). All samples were provided through the Department of Surgery and the Department of Dermatology and Skin Science, University of British Columbia, with approval from the University Clinical Research Ethics Board. All participants gave written informed consent before participation in the study.

HF (n = 10−20 per sample group per subject) were microdissected partly using the method of Philpott and colleagues [39]. In brief, punch biopsies into the subcutaneous fat were performed, yielding 4-mm cylindrical cores of tissue. A scalpel blade was used to cut through the skin at the epidermis-dermis interface. The dermal sheaths of the HFs were gently gripped and whole HFs were pulled out from the remaining tissue with forceps; both pigmented and non-pigmented HFs were taken from each biopsy. Any remaining tissue surrounding the HFs was removed using needles. The intact HFs, including hair shafts, were isolated without sustaining any visible damage and collected in separate pools for parallel processing.

**Figure 5:**
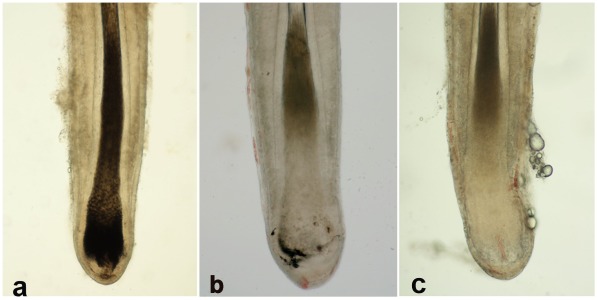
Pigmented and non–pigmented microdissected human hair follicles. Hair follicles were separated into sample groups; those with pigmentation, where the dermal papilla was hidden in a strongly pigmented hair bulb and pigment-presence was evident in the hair shaft (a). The few hair follicles with apparent pigmentation dilution, but not fully devoid of pigmentation, were discarded (b); and those where no pigmented melanocytes could be observed macroscopically in the hair bulb and the hair shaft appeared as fully unpigmented white hair (c).

Anagen stage HF were separated into two sample groups; those with pigmentation, where the dermal papilla was hidden in a strongly pigmented HB and pigment-activity was evident in the hair shaft; and those where macroscopically no pigmented melanocytes could be detected in the HB and the hair shaft appeared fully unpigmented. HFs with apparent pigmentation dilution, but not fully devoid of pigmentation (Fig. 5), were very few and consequently could not be included in this study. Pigmented and non-pigmented HFs were then sub-divided for analysis into the lower one third, including the HB, and into the keratinized hair shaft and root sheaths, including the bulge region.

### RNA Isolation and Microarray Processing

Total RNA was isolated from microdissected tissue with an RNeasy Fibrous Tissue Midi Kit (Qiagen, Mississauga, ON, Canada) according to the manufacturer’s protocols. RNA quality and the quantity were measured using the Agilent 2100 bioanalyzer (Agilent Technologies, Palo Alto, CA). Human Operon v.2.1 (21 K) glass arrays were produced (based on human 70mers from Operon Biotechnologies Inc, Huntsville, AL) by the Microarray Facility of the Prostate Centre at Vancouver General Hospital, Vancouver, Canada [40]. RNAs were amplified using the SenseAmp Plus kit (Genisphere Inc, Hatfield, PA). Total RNA from test samples and universal human reference RNA (Stratagene, Cedar Creek, TX) were respectively labeled with Cy5 and Cy3, using the 3DNA array detection 350 kit (Genisphere), cohybridized to cDNA microarrays, and imaged using a ScanArray Express scanner (PerkinElmer, Boston, MA).

### Data Processing and Analysis

All microarray data processing and analysis are compliance to MIAME guidelines. All raw data from the arrays are publicly accessible in the Gene Expression Omnibus (GEO) database (http://www.ncbi.nlm.nih.gov/geo/) encompassed by the series number (GSE24009). The raw data were inputted into Genespring 7.2 program (Silicon Genetics, Redwood City, CA) and analyzed using protocols detailed elsewhere [41]. Briefly, unsupervised hierarchical clustering analysis was applied to the unfiltered datasets from HS and HB samples. Similarity was measured using Pearson Correlation and distances between clusters were calculated via average linkage. Pigmented HS, non-pigmented HS, and pigmented HBs were fairly similar to each other. However, the gene expression profiles of non pigmented HBs were much more variable and could not be easily associated with each other (data not shown). The differences analysis between pigmented and non-pigmented HS and HBs, were conducted by the SAM method [42] with a cut-off *q*-value and a 2 fold cut-off (Table S1and Table S2). The cut above 2 fold reduced the chance of false positives which can occur using t-tests (replicates having similar results by chance). The probability for this occurrence decreases at higher fold changes.

### Gene Ontology Analysis

Functional classification of genes using DAVID software, based on the Gene Ontology (GO) database (www.geneontology.org), identified “enriched” or “depleted” gene function categories [7]. A Benjamini-corrected p<0.05 was used as the cutoff for determination of significant gene enrichment. A P-Value < = 0.05, indicated the applied genes listed were specifically associated in a category as compared to random chance. Ontological analyses were performed at biological process category level 3. The numbers of genes identified in major categories were normalized by the number of genes annotated in each list and were expressed as percentages (Table S3 and Table S4).

### Real-time Reverse Transcription-PCR Analysis for Gene Expression

To verify the alterations in gene expression at the mRNA level identified in microarray results, we chose 11 representative genes from the NER pathway for quantitative PCR analysis (Fig. 1) using protocols described elsewhere [41]. Primer sequences are listed (Table S5).

### Immunohistology

Immunofluorescent detection of ERCC3-immunoreactive cells was performed with anti-ERCC3 monoclonal antibody (MAb) (No: ab85408; Abcam, Cambridge, MA) and melanocyte marker, gp100 (No: M0634, Dako, Mississauga, ON). The pre-melanosomal marker gp100 [43] was included for its reactivity with both mature melanocytes and premature melanocytes in the ORS which contain early structural proteins (premelanosomes), but not later structural proteins (melanosomes) or enzymatic proteins, tyrosinase, TRP-1, or TRP-2 proteins [23], [43], [44], [45], [46]. Double labeling was done on formalin-fixed, paraffin-embedded normal scalp and pigmented nevi biopsies (tissues from 4 individuals per group). Briefly, immunofluorescence labeling was undertaken on these sections following deparaffinization, and heating in a water bath to 95°C for 30 min in antigen target retrieval solution (No: S1699, Dako, Mississauga, ON) to retrieve the antigen. The sections were incubated with goat serum followed by incubation with anti-ERCC3 and anti-gp100 primary antibodies overnight at 4°C. After extensive washing, slides were incubated at room temperature with red fluorescent Alexa Fluor 594 dye-labeled anti-rabbit IgG (No: A31620, Invitrogen, Canada) for ERCC3 and green-fluorescent Alexa Fluor 488 dye-labeled anti-mouse IgG (A31632, Invitrogen Canada) for gp100. The slides were mounted in antifading mounting medium with 4′, 6-diamidino-2-phenylindole (DAPI; Vectashield; Vector Laboratories Inc., Burlingame, CA). Slides were observed under a fluorescent microscope with corresponding filters. Pictures were captured by a digital camera system. Sections incubated without primary or secondary antibody were used as negative controls to verify the lack of autofluorescence and nonspecific secondary antibody staining.

### Cell Lines, Cell Culture and siRNA Transfection

Although, there are some behavioral differences between adult and neonatal melanocytes in culture, there are no age-dependent differences in melanin content and cell proliferation rates between neonatal and adult cultured melanocytes. Neonatal melanocytes are considered an acceptable model for studying the mechanisms of adult-onset hyperpigmentary disorders and melanogenesis [47]. We chose Human Epidermal Melanocyte, neonatal cells from darkly pigmented neonatal foreskin (HEMn-DP) (Invitrogen, Burlington, ON, Canada) for our *in vitro* study. Cells were maintained in Medium 254 supplemented with human melanocyte growth supplement (Invitrogen). Cells were plated at a density of 2.5 to 5×10^5^ cells/ml. Using PromoFectin-siRNA transfection reagent (Promokine, Heidelberg, Germany), cells were transfected with either ON-TARGET*plus* Non-Targeting Pool (50 nM) (D-001810-10, Dharmacon, Inc., Lafayette, CO) or ON-TARGET *plus* Smart pool *ERCC3* siRNA (50 nM) (L-011028-00-0020, Human ERCC3, NM-000122, Dharmacon); 24 hours after transfection, cells were collected. Cell numbers were counted before and after transfection.

### Tyrosinase Assay

Tyrosinase activity was spectrophotometrically assayed as DOPA oxidase activity using a previously reported method [21]. Assays were carried out at 37°C in a spectrophotometer and the absorbance at 470 nm was read at 0, 20, 40 and 60 min per cell sample.

## Supporting Information

Table S1 **Gene transcripts with significant upregulation (a) and downregulation (b) in non-pigmented hair bulb versus pigmented hair bulb.** Top twenty five genes sorted by the false discovery rate (q-value).
(DOC)

Table S2 **Gene transcripts with significant upregulation (a) and downregulation (b) in non-pigmented hair bulb versus pigmented hair bulb.** Top twenty five genes sorted by the false discovery rate (q-value).
(DOC)

Table S3 **Differences in Gene Ontology categorization analysis between non-pigmented hair bulb versus pigmented hair bulb at GO level 3.** All categories identified are shown.
(DOC)

Table S4 **Differences in Gene Ontology categorization analysis between non-pigmented hair sheath versus pigmented hair sheath at GO level 3.** All categories identified are shown.
(DOC)

Table S5 **Primer sequences for defined genes.**
(DOC)

## References

[1] Stenn KS, Paus R (2001). Controls of hair follicle cycling.. Physiol Rev.

[2] Commo S, Bernard BA (2000). Melanocyte subpopulation turnover during the human hair cycle: an immunohistochemical study.. Pigment Cell Res.

[3] Tobin DJ, Paus R (2001). Graying: gerontobiology of the hair follicle pigmentary unit.. Exp Gerontol.

[4] Cotsarelis G (2006). Epithelial stem cells: a folliculocentric view.. J Invest Dermatol.

[5] Steingrimsson E, Copeland NG, Jenkins NA (2005). Melanocyte stem cell maintenance and hair graying.. Cell.

[6] Rabbani P, Takeo M, Chou W, Myung P, Bosenberg M (2011). Coordinated activation of Wnt in epithelial and melanocyte stem cells initiates pigmented hair regeneration.. Cell.

[7] Harris MA, Clark J, Ireland A, Lomax J, Ashburner M (2004). The Gene Ontology (GO) database and informatics resource.. Nucleic Acids Res.

[8] Sarin KY, Artandi SE (2007). Aging, graying and loss of melanocyte stem cells.. Stem Cell Rev.

[9] Tobin DJ, Hagen E, Botchkarev VA, Paus R (1998). Do hair bulb melanocytes undergo apoptosis during hair follicle regression (catagen)?. J Invest Dermatol.

[10] Slominski A, Paus R (1993). Melanogenesis is coupled to murine anagen: toward new concepts for the role of melanocytes and the regulation of melanogenesis in hair growth.. J Invest Dermatol.

[11] Nishimura EK, Jordan SA, Oshima H, Yoshida H, Osawa M (2002). Dominant role of the niche in melanocyte stem-cell fate determination.. Nature.

[12] Van Neste D, Tobin DJ (2004). Hair cycle and hair pigmentation: dynamic interactions and changes associated with aging.. Micron.

[13] Slominski A, Tobin DJ, Shibahara S, Wortsman J (2004). Melanin pigmentation in mammalian skin and its hormonal regulation.. Physiol Rev.

[14] Arck PC, Overall R, Spatz K, Liezman C, Handjiski B (2006). Towards a “free radical theory of graying”: melanocyte apoptosis in the aging human hair follicle is an indicator of oxidative stress induced tissue damage.. Faseb J.

[15] Nishimura EK, Granter SR, Fisher DE (2005). Mechanisms of hair graying: incomplete melanocyte stem cell maintenance in the niche.. Science.

[16] Inomata K, Aoto T, Binh NT, Okamoto N, Tanimura S (2009). Genotoxic stress abrogates renewal of melanocyte stem cells by triggering their differentiation.. Cell.

[17] Green ML, Karp PD (2006). The outcomes of pathway database computations depend on pathway ontology.. Nucleic Acids Res.

[18] Etienne W, Meyer MH, Peppers J, Meyer RA (2004). Comparison of mRNA gene expression by RT-PCR and DNA microarray.. Biotechniques 36: 618–620, 622,.

[19] Dallas PB, Gottardo NG, Firth MJ, Beesley AH, Hoffmann K (2005). Gene expression levels assessed by oligonucleotide microarray analysis and quantitative real-time RT-PCR – how well do they correlate?. BMC Genomics.

[20] Kim DS, Hwang ES, Lee JE, Kim SY, Kwon SB (2003). Sphingosine-1-phosphate decreases melanin synthesis via sustained ERK activation and subsequent MITF degradation.. J Cell Sci.

[21] Ando H, Funasaka Y, Oka M, Ohashi A, Furumura M (1999). Possible involvement of proteolytic degradation of tyrosinase in the regulatory effect of fatty acids on melanogenesis.. J Lipid Res.

[22] Passeron T, Coelho SG, Miyamura Y, Takahashi K, Hearing VJ (2007). Immunohistochemistry and in situ hybridization in the study of human skin melanocytes.. Exp Dermatol.

[23] Tobin DJ, Hordinsky M, Bernard BA (2005). Hair pigmentation: a research update.. J Investig Dermatol Symp Proc.

[24] Cui J, Shen LY, Wang GC (1991). Role of hair follicles in the repigmentation of vitiligo.. J Invest Dermatol.

[25] Tanimura S, Tadokoro Y, Inomata K, Binh NT, Nishie W (2011). Hair follicle stem cells provide a functional niche for melanocyte stem cells.. Cell Stem Cell.

[26] Isken O, Maquat LE (2008). The multiple lives of NMD factors: balancing roles in gene and genome regulation.. Nat Rev Genet.

[27] Mitchell JR, Hoeijmakers JH, Niedernhofer LJ (2003). Divide and conquer: nucleotide excision repair battles cancer and ageing.. Curr Opin Cell Biol.

[28] Cooke MS, Evans MD, Dizdaroglu M, Lunec J (2003). Oxidative DNA damage: mechanisms, mutation, and disease.. Faseb J.

[29] de Laat WL, Jaspers NG, Hoeijmakers JH (1999). Molecular mechanism of nucleotide excision repair.. Genes Dev.

[30] Weeda G, Eveno E, Donker I, Vermeulen W, Chevallier-Lagente O (1997). A mutation in the XPB/ERCC3 DNA repair transcription gene, associated with trichothiodystrophy.. Am J Hum Genet.

[31] Beck BD, Hah DS, Lee SH (2008). XPB and XPD between transcription and DNA repair.. Adv Exp Med Biol.

[32] de Boer J, Andressoo JO, de Wit J, Huijmans J, Beems RB (2002). Premature aging in mice deficient in DNA repair and transcription.. Science.

[33] Andressoo JO, Weeda G, de Wit J, Mitchell JR, Beems RB (2009). An Xpb mouse model for combined xeroderma pigmentosum and cockayne syndrome reveals progeroid features upon further attenuation of DNA repair.. Mol Cell Biol.

[34] Eller MS, Maeda T, Magnoni C, Atwal D, Gilchrest BA (1997). Enhancement of DNA repair in human skin cells by thymidine dinucleotides: evidence for a p53-mediated mammalian SOS response.. Proc Natl Acad Sci U S A.

[35] Egly JM (2001). The 14th Datta Lecture. TFIIH: from transcription to clinic.. FEBS Lett.

[36] Le Page F, Kwoh EE, Avrutskaya A, Gentil A, Leadon SA (2005). Transcription-coupled repair of 8-oxoguanine: requirement for XPG, TFIIH, and CSB and implications for Cockayne syndrome.. Cell.

[37] de Boer J, de Wit J, van Steeg H, Berg RJ, Morreau H (1998). A mouse model for the basal transcription/DNA repair syndrome trichothiodystrophy.. Mol Cell.

[38] Gilchrest BA, Bohr VA (1997). Aging processes, DNA damage, and repair.. Faseb J.

[39] Philpott MP, Green MR, Kealey T (1990). Human hair growth in vitro.. J Cell Sci 97 (Pt.

[40] Nelson CC, Hoffart D, Gleave ME, Rennie PS (2003). Application of gene microarrays in the study of prostate cancer.. Methods Mol Med.

[41] Yu M, Zloty D, Cowan B, Shapiro J, Haegert A (2008). Superficial, nodular, and morpheiform basal-cell carcinomas exhibit distinct gene expression profiles.. J Invest Dermatol.

[42] Tusher VG, Tibshirani R, Chu G (2001). Significance analysis of microarrays applied to the ionizing radiation response.. Proc Natl Acad Sci U S A.

[43] Kim D, Kang WH (2002). Role of dermal melanocytes in cutaneous pigmentation of stasis dermatitis: a histopathological study of 20 cases.. J Korean Med Sci.

[44] Gown AM, Vogel AM (1982). Monoclonal antibodies to intermediate filament proteins of human cells: unique and cross-reacting antibodies.. J Cell Biol.

[45] Kapur RP, Bigler SA, Skelly M, Gown AM (1992). Anti-melanoma monoclonal antibody HMB45 identifies an oncofetal glycoconjugate associated with immature melanosomes.. J Histochem Cytochem.

[46] Esclamado RM, Gown AM, Vogel AM (1986). Unique proteins defined by monoclonal antibodies specific for human melanoma. Some potential clinical applications.. Am J Surg.

[47] Kim NS, Cho JH, Kang WH (2000). Behavioral differences between donor site-matched adult and neonatal melanocytes in culture.. Arch Dermatol Res.

