# Supplementary material for: Deficiency in Nucleotide Excision Repair Family Gene Activity, Especially ERCC3, Is Associated with Non-Pigmented Hair Fiber Growth
Source: PLoS One. 2012 May 16;7(5):e34185. doi: 10.1371/journal.pone.0034185 (PMC3353974; doi:10.1371/journal.pone.0034185)
Supplement: Table S1 — Gene transcripts with significant upregulation (a) and downregulation (b) in non-pigmented hair bulb versus pigmented hair bulb. Top twenty five genes sorted by the false discovery rate (q-value). (DOC) [file pone.0034185.s001.doc]

**Supporting Information S1**

***Table S1a. Gene transcripts with significant upregulation in non-pigmented hair bulb versus pigmented hair bulb***

| **Gene Name** | **Gene Symbol** | **GenBank Accession Number** | **Fold Change** | **Q-value (%)** |
| --- | --- | --- | --- | --- |
| KIAA1317 protein | KIAA1317 | [AB037738](http://genome-www4.stanford.edu/cgi-bin/SMD/source/sourceResult?choice=Gene&option=Name&criteria=AB037738) | 131.8497 | 100 |
| Retinoblastoma binding protein 5 | RBBP5 | [NM_005057](http://genome-www4.stanford.edu/cgi-bin/SMD/source/sourceResult?choice=Gene&option=Name&criteria=NM_005057) | 99.13451 | 100 |
| Uncharacterized hypothalamus protein HT009 | HT009 | [NM_018470](http://genome-www4.stanford.edu/cgi-bin/SMD/source/sourceResult?choice=Gene&option=Name&criteria=NM_018470) | 83.17397 | 100 |
| Class II cytokine receptor | IL22RA2 | [NM_052962](http://genome-www4.stanford.edu/cgi-bin/SMD/source/sourceResult?choice=Gene&option=Name&criteria=NM_052962) | 79.01982 | 100 |
| Carbonic anhydrase-related protein 10 | CARPX | [NM_020178](http://genome-www4.stanford.edu/cgi-bin/SMD/source/sourceResult?choice=Gene&option=Name&criteria=NM_020178) | 64.58458 | 100 |
| KIAA1735 protein | KIAA1735 | [AB051522](http://genome-www4.stanford.edu/cgi-bin/SMD/source/sourceResult?choice=Gene&option=Name&criteria=AB051522) | 59.00953 | 100 |
| MD-1, RP105-associated | MD-1 | [NM_004271](http://genome-www4.stanford.edu/cgi-bin/SMD/source/sourceResult?choice=Gene&option=Name&criteria=NM_004271) | 49.04436 | 100 |
| Centrin, EF-hand protein, 1 | CETN1 | [NM_004066](http://genome-www4.stanford.edu/cgi-bin/SMD/source/sourceResult?choice=Gene&option=Name&criteria=NM_004066) | 42.79291 | 100 |
| FK506-binding protein | LOC51661 | [AF092137](http://genome-www4.stanford.edu/cgi-bin/SMD/source/sourceResult?choice=Gene&option=Name&criteria=AF092137) | 41.19967 | 100 |
| KIAA0467 protein | KIAA0467 | [AB007936](http://genome-www4.stanford.edu/cgi-bin/SMD/source/sourceResult?choice=Gene&option=Name&criteria=AB007936) | 38.29394 | 100 |
| ELAV (embryonic lethal, abnormal vision, Drosophila)-like 4 (Hu antigen D) | ELAVL4 | [NM_021952](http://genome-www4.stanford.edu/cgi-bin/SMD/source/sourceResult?choice=Gene&option=Name&criteria=NM_021952) | 32.91936 | 100 |
| Hypothetical protein FLJ23059 | FLJ23059 | [NM_032234](http://genome-www4.stanford.edu/cgi-bin/SMD/source/sourceResult?choice=Gene&option=Name&criteria=NM_032234) | 32.90488 | 100 |
| Retinoic acid receptor responder (tazarotene induced) 1 | RARRES1 | [NM_002888](http://genome-www4.stanford.edu/cgi-bin/SMD/source/sourceResult?choice=Gene&option=Name&criteria=NM_002888) | 29.97442 | 100 |
| UDP-N-acetyl-alpha-D-galactosamine:polypeptide N-acetylgalactosaminyltransferase 9 (GalNAc-T9) | GALNT9 | [NM_021808](http://genome-www4.stanford.edu/cgi-bin/SMD/source/sourceResult?choice=Gene&option=Name&criteria=NM_021808) | 24.59658 | 100 |
| KIAA0552 gene product | KIAA0552 | [NM_014731](http://genome-www4.stanford.edu/cgi-bin/SMD/source/sourceResult?choice=Gene&option=Name&criteria=NM_014731) | 22.87885 | 100 |
| Pancreatic beta cell growth factor | INGAP | [NM_012277](http://genome-www4.stanford.edu/cgi-bin/SMD/source/sourceResult?choice=Gene&option=Name&criteria=NM_012277) | 20.54803 | 100 |
| Insulin-like 5 | INSL5 | [NM_005478](http://genome-www4.stanford.edu/cgi-bin/SMD/source/sourceResult?choice=Gene&option=Name&criteria=NM_005478) | 18.01652 | 100 |
| Histone deacetylase 7A | HDAC7A | [NM_015401](http://genome-www4.stanford.edu/cgi-bin/SMD/source/sourceResult?choice=Gene&option=Name&criteria=NM_015401) | 17.77781 | 100 |
| KIAA0980 protein | KIAA0980 | [AB023197](http://genome-www4.stanford.edu/cgi-bin/SMD/source/sourceResult?choice=Gene&option=Name&criteria=AB023197) | 17.62618 | 100 |
| Uroplakin 2 | UPK2 | [NM_006760](http://genome-www4.stanford.edu/cgi-bin/SMD/source/sourceResult?choice=Gene&option=Name&criteria=NM_006760) | 17.45209 | 100 |
| KIAA0318 protein | KIAA0318 | [AB002316](http://genome-www4.stanford.edu/cgi-bin/SMD/source/sourceResult?choice=Gene&option=Name&criteria=AB002316) | 14.20365 | 100 |
| Apolipoprotein F | APOF | [NM_001638](http://genome-www4.stanford.edu/cgi-bin/SMD/source/sourceResult?choice=Gene&option=Name&criteria=NM_001638) | 14.04146 | 100 |
| Carbonic anhydrase VA, mitochondrial | CA5A | [NM_001739](http://genome-www4.stanford.edu/cgi-bin/SMD/source/sourceResult?choice=Gene&option=Name&criteria=NM_001739) | 13.76756 | 100 |
| C-Mpl binding protein | LOC113251 | [NM_052879](http://genome-www4.stanford.edu/cgi-bin/SMD/source/sourceResult?choice=Gene&option=Name&criteria=NM_052879) | 13.04099 | 100 |
| Hypothetical protein FLJ10706 | FLJ10706 | [NM_018186](http://genome-www4.stanford.edu/cgi-bin/SMD/source/sourceResult?choice=Gene&option=Name&criteria=NM_018186) | 13.00872 | 100 |

Top twenty five genes sorted by the false discovery rate (q-value).

***Table S1b. Gene transcripts with significant downregulation in non-pigmented hair bulb versus pigmented hair bulb***

| **Gene Name** | **Gene Symbol** | **GenBank Accession Number** | **Fold Change** | **Q-value (%)** |
| --- | --- | --- | --- | --- |
| Melan-A | MLANA | NM_005511 | 0.060407093 | 0 |
| Oculospanin | OCSP | NM_031945 | 0.21279367 | 0 |
| Kell blood group precursor (McLeod phenotype) | XK | NM_021083 | 0.500153707 | 0 |
| Ras homolog gene family, member A | ARHA | NM_001664 | 0.651201449 | 24.45784766 |
| Splicing factor, arginine/serine-rich, 46kD | SRP46 | BC006181 | 0.676184901 | 24.45784766 |
| Trophoblast glycoprotein | TPBG | NM_006670 | 0.481090267 | 39.13255625 |
| Tyrosinase-related protein 1 | TYRP1 | NM_000550 | 0.185263258 | 39.13255625 |
| Heat shock 90kD protein 1, beta | HSPCB | NM_007355 | 0.553663499 | 39.13255625 |
| Solute carrier family 2 (facilitated glucose transporter), member 1 | SLC2A1 | NM_006516 | 0.814355342 | 39.13255625 |
| Zinc finger protein | ZF5128 | NM_014347 | 0.086793539 | 39.13255625 |
| Ribosomal protein S28 | RPS28 | NM_001031 | 0.517524327 | 48.91569531 |
| 5-hydroxytryptamine (serotonin) receptor 6 | HTR6 | NM_000871 | 0.505382355 | 48.91569531 |
| Membrane-associated tyrosine- and threonine-specific cdc2-inhibitory kinase | PKMYT1 | NM_004203 | 0.705707028 | 48.91569531 |
| SRY (sex determining region Y)-box 18 | SOX18 | NM_018419 | 0.361520195 | 53.80726484 |
| Major histocompatibility complex, class I, A | HLA-A | NM_002116 | 0.478066306 | 53.80726484 |
| Tumor-associated calcium signal transducer 2 | TACSTD2 | NM_002353 | 0.531379661 | 53.80726484 |
| Potassium voltage-gated channel, shaker-related subfamily, member 2 | KCNA2 | NM_004974 | 0.488526243 | 53.80726484 |
| HSPC166 protein | HSPC166 | NM_014186 | 0.644031515 | 53.80726484 |
| Cylindromatosis (turban tumor syndrome) | CYLD | NM_015247 | 0.0919593 | 53.80726484 |
| Translocase of outer mitochondrial membrane 20 (yeast) homolog | KIAA0016 | NM_014765 | 0.147215261 | 53.80726484 |
| Hypothetical protein FLJ22233 | FLJ22233 | NM_024959 | 0.238510261 | 53.80726484 |
| Defender against cell death 1 | DAD1 | NM_001344 | 0.713589113 | 53.80726484 |
| Ribonuclease P (30kD) | RPP30 | BC006991 | 0.117609717 | 53.80726484 |
| G protein-coupled receptor, family C, group 1, member B | GPRC5B | NM_016235 | 0.654662181 | 53.80726484 |

Top twenty five genes sorted by the false discovery rate (q-value).
