# Supplementary material for: Deficiency in Nucleotide Excision Repair Family Gene Activity, Especially ERCC3, Is Associated with Non-Pigmented Hair Fiber Growth
Source: PLoS One. 2012 May 16;7(5):e34185. doi: 10.1371/journal.pone.0034185 (PMC3353974; doi:10.1371/journal.pone.0034185)
Supplement: Table S2 — Gene transcripts with significant upregulation (a) and downregulation (b) in non-pigmented hair bulb versus pigmented hair bulb. Top twenty five genes sorted by the false discovery rate (q-value). (DOC) [file pone.0034185.s002.doc]

**Supporting information S2**

***Table S2a. Gene transcripts with significant upregulation in non- pigmented hair sheath versus pigmented hair sheath***

| **Gene Name** | **Gene Symbol** | **GenBank Accession Number** | **Fold Change** | **Q-value (%)** |
| --- | --- | --- | --- | --- |
| Solute carrier family 26, member 9 | SLC26A9 | [NM_052934](http://genome-www4.stanford.edu/cgi-bin/SMD/source/sourceResult?choice=Gene&option=Name&criteria=NM_052934) | 175.20745 | 0 |
| Down syndrome critical region gene 3 | DSCR3 | [NM_006052](http://genome-www4.stanford.edu/cgi-bin/SMD/source/sourceResult?choice=Gene&option=Name&criteria=NM_006052) | 108.33371 | 0 |
| KIAA1821 protein | KIAA1821 | [AB058724](http://genome-www4.stanford.edu/cgi-bin/SMD/source/sourceResult?choice=Gene&option=Name&criteria=AB058724) | 40.5651285 | 0 |
| PRO0149 protein | PRO0149 | [NM_014117](http://genome-www4.stanford.edu/cgi-bin/SMD/source/sourceResult?choice=Gene&option=Name&criteria=NM_014117) | 379.1338101 | 16.87599771 |
| CAP-binding protein complex interacting protein 1 | HSCBCIP1 | [AK058069](http://genome-www4.stanford.edu/cgi-bin/SMD/source/sourceResult?choice=Gene&option=Name&criteria=AK058069) | 150.3081224 | 73.64071728 |
| Putative selenocysteine lyase | SCLY | [AK002119](http://genome-www4.stanford.edu/cgi-bin/SMD/source/sourceResult?choice=Gene&option=Name&criteria=AK002119) | 18.7423805 | 73.64071728 |
| KIAA0390 gene product | KIAA0390 | [NM_014717](http://genome-www4.stanford.edu/cgi-bin/SMD/source/sourceResult?choice=Gene&option=Name&criteria=NM_014717) | 18.52456554 | 73.64071728 |
| DKFZP586G1122 protein | DKFZP586G1122 | [AF304052](http://genome-www4.stanford.edu/cgi-bin/SMD/source/sourceResult?choice=Gene&option=Name&criteria=AF304052) | 9.99246289 | 73.64071728 |
| Uncharacterized hematopoietic stem/progenitor cells protein MDS032 | MDS032 | [NM_018467](http://genome-www4.stanford.edu/cgi-bin/SMD/source/sourceResult?choice=Gene&option=Name&criteria=NM_018467) | 6.640021265 | 73.64071728 |
| Diazepam binding inhibitor (GABA receptor modulator, acyl-Coenzyme A binding protein) | DBI | [NM_020548](http://genome-www4.stanford.edu/cgi-bin/SMD/source/sourceResult?choice=Gene&option=Name&criteria=NM_020548) | 4.847401904 | 73.64071728 |
| Serine/threonine kinase 18 | STK18 | [NM_014264](http://genome-www4.stanford.edu/cgi-bin/SMD/source/sourceResult?choice=Gene&option=Name&criteria=NM_014264) | 109.183023 | 82.84580694 |
| C-Mpl binding protein | LOC113251 | [NM_052879](http://genome-www4.stanford.edu/cgi-bin/SMD/source/sourceResult?choice=Gene&option=Name&criteria=NM_052879) | 106.6312992 | 82.84580694 |
| Claudin 12 | CLDN12 | [AL136770](http://genome-www4.stanford.edu/cgi-bin/SMD/source/sourceResult?choice=Gene&option=Name&criteria=AL136770) | 104.208017 | 82.84580694 |
| Protease inhibitor 15 | PI15 | [NM_015886](http://genome-www4.stanford.edu/cgi-bin/SMD/source/sourceResult?choice=Gene&option=Name&criteria=NM_015886) | 100.93308 | 82.84580694 |
| DEAD/H (Asp-Glu-Ala-Asp/His) box polypeptide 28 | DDX28 | [NM_018380](http://genome-www4.stanford.edu/cgi-bin/SMD/source/sourceResult?choice=Gene&option=Name&criteria=NM_018380) | 83.591152 | 82.84580694 |
| Toll-like receptor 5 | TLR5 | [NM_003268](http://genome-www4.stanford.edu/cgi-bin/SMD/source/sourceResult?choice=Gene&option=Name&criteria=NM_003268) | 77.31626968 | 82.84580694 |
| Hypothetical protein FLJ14075 | FLJ14075 | [NM_024894](http://genome-www4.stanford.edu/cgi-bin/SMD/source/sourceResult?choice=Gene&option=Name&criteria=NM_024894) | 60.722094 | 82.84580694 |
| Natriuretic peptide receptor A/guanylate cyclase A (atrionatriuretic peptide receptor A) | NPR1 | [NM_000906](http://genome-www4.stanford.edu/cgi-bin/SMD/source/sourceResult?choice=Gene&option=Name&criteria=NM_000906) | 45.0954035 | 82.84580694 |
| KIAA0999 protein | KIAA0999 | [AB023216](http://genome-www4.stanford.edu/cgi-bin/SMD/source/sourceResult?choice=Gene&option=Name&criteria=AB023216) | 43.397124 | 82.84580694 |
| Excision repair cross-complementing rodent repair deficiency, complementation group 6 | ERCC6 | [NM_000124](http://genome-www4.stanford.edu/cgi-bin/SMD/source/sourceResult?choice=Gene&option=Name&criteria=NM_000124) | 39.512195 | 82.84580694 |
| B-cell translocation gene 4 | BTG4 | [NM_017589](http://genome-www4.stanford.edu/cgi-bin/SMD/source/sourceResult?choice=Gene&option=Name&criteria=NM_017589) | 38.429693 | 82.84580694 |
| Vitelliform macular dystrophy (Best disease, bestrophin) | VMD2 | NM_004183 | 35.364239 | 82.84580694 |
| AF15q14 protein | AF15Q14 | NM_020380 | 30.48090933 | 82.84580694 |
| CocoaCrisp | LOC83690 | NM_031461 | 27.331706 | 82.84580694 |
| Sarcoglycan | SGCA | NM_000023 | 26.6251925 | 82.84580694 |

Top twenty five genes sorted by the false discovery rate (q-value).

***Table S2b. Gene transcripts with significant downregulation in non-pigmented hair sheath versus pigmented hair sheath***

| **Gene Name** | **Gene Symbol** | **GenBank Accession Number** | **Fold Change** | **Q-value (%)** |
| --- | --- | --- | --- | --- |
| Hypothetical protein FLJ20285 | FLJ20285 | NM_017745 | 0.480527 | 73.64072 |
| Tomosyn | STXBP-TOM | AB023223 | 0.000316 | 98.44938 |
| Protocadherin alpha subfamily C, 2 | PCDHAC2 | NM_018899 | 0.000493 | 98.44938 |
| Novel protein | HSNOV1 | NM_017515 | 0.000653 | 98.44938 |
| GREB1 protein | GREB1 | AF245389 | 0.000953 | 98.44938 |
| KIAA1493 protein | KIAA1493 | AB040926 | 0.001281 | 98.44938 |
| Phospholipid scramblase 2 | PLSCR2 | NM_020359 | 0.001341 | 98.44938 |
| X 010 protein | MDS010 | NM_020231 | 0.001397 | 98.44938 |
| PRO1600 protein | PRO1600 | NM_014095 | 0.001449 | 98.44938 |
| Hypothetical protein FLJ13166 | FLJ13166 | NM_025003 | 0.001585 | 98.44938 |
| Williams-Beuren syndrome chromosome region 23 | WBSCR23 | NM_025042 | 0.001676 | 98.44938 |
| Homo sapiens mRNA; cDNA DKFZp564N2464 (from clone DKFZp564N2464) | N/A | AL137578 | 0.001889 | 98.44938 |
| Ig superfamily protein | Z39IG | NM_007268 | 0.002034 | 98.44938 |
| Hypothetical protein FLJ14345 | FLJ14345 | NM_024733 | 0.002081 | 98.44938 |
| Homo sapiens mRNA; cDNA DKFZp434I0623 (from clone DKFZp434I0623) | N/A | AL137476 | 0.002305 | 98.44938 |
| Acetylcholinesterase (YT blood group) | ACHE | NM_015831 | 0.002409 | 98.44938 |
| Hypothetical protein | FLJ20225 | NM_019062 | 0.002431 | 98.44938 |
| Oxidised low density lipoprotein (lectin-like) receptor 1 | OLR1 | NM_002543 | 0.002884 | 98.44938 |
| Netrin G1 | KIAA0976 | NM_014917 | 0.002908 | 98.44938 |
| FLJ00005 protein | FLJ00005 | AK000005 | 0.002954 | 98.44938 |
| Cell adhesion molecule with homology to L1CAM (close homolog of L1) | CHL1 | NM_006614 | 0.002958 | 98.44938 |
| Homo sapiens mRNA; cDNA DKFZp564I1316 (from clone DKFZp564I1316) | N/A | AL110127 | 0.002989 | 98.44938 |
| Prostaglandin F receptor (FP) | PTGFR | NM_000959 | 0.00302 | 98.44938 |
| Mucosa associated lymphoid tissue lymphoma translocation gene 1 | MALT1 | NM_006785 | 0.00305 | 98.44938 |
| Mannan-binding lectin serine protease 1 (C4/C2 activating component of Ra-reactive factor) | MASP1 | D17525 | 0.003075 | 98.44938 |
