# Supplementary material for: Deficiency in Nucleotide Excision Repair Family Gene Activity, Especially ERCC3, Is Associated with Non-Pigmented Hair Fiber Growth
Source: PLoS One. 2012 May 16;7(5):e34185. doi: 10.1371/journal.pone.0034185 (PMC3353974; doi:10.1371/journal.pone.0034185)
Supplement: Table S3 — Differences in Gene Ontology categorization analysis between non-pigmented hair bulb versus pigmented hair bulb at GO level 3. All categories identified are shown. (DOC) [file pone.0034185.s003.doc]

**Supporting Information S3**

***Table S3. Differences in Gene Ontology categorization analysis between non-pigmented hair bulb versus pigmented hair bulb at GO level 3***

| **GO category** | **Number of genes in Category** | **Percentage of total genes in gene set** | **P-Value** | **FDR** |
| --- | --- | --- | --- | --- |
| gene expression | 412 | 19.29742 | 1.23E-05 | 0.019103781 |
| cellular macromolecule metabolic process | 674 | 31.56909 | 2.33E-05 | 0.036247786 |
| macromolecule biosynthetic process | 383 | 17.93911 | 1.18E-04 | 0.182829922 |
| cellular biosynthetic process | 455 | 21.31148 | 1.85E-04 | 0.287489318 |
| cellular nitrogen compound metabolic process | 478 | 22.38876 | 4.73E-04 | 0.732621562 |
| nucleobase, nucleoside, nucleotide and nucleic acid metabolic process | 446 | 20.88993 | 5.55E-04 | 0.858996711 |
| negative regulation of cellular metabolic process | 110 | 5.152225 | 0.00137 | 2.108296018 |
| mitotic cell cycle | 61 | 2.857143 | 0.003534 | 5.354704728 |
| positive regulation of metabolic process | 133 | 6.229508 | 0.003577 | 5.417650758 |
| negative regulation of macromolecule metabolic process | 109 | 5.105386 | 0.003681 | 5.571115004 |
| negative regulation of metabolic process | 114 | 5.339578 | 0.00499 | 7.481159451 |
| cell cycle process | 86 | 4.028103 | 0.005272 | 7.888532374 |
| positive regulation of macromolecule metabolic process | 123 | 5.761124 | 0.006299 | 9.354885541 |
| positive regulation of biosynthetic process | 102 | 4.777518 | 0.00701 | 10.35856266 |
| cellular macromolecule localization | 65 | 3.044496 | 0.007957 | 11.67815283 |
| positive regulation of nitrogen compound metabolic process | 95 | 4.449649 | 0.008276 | 12.11807655 |
| negative regulation of cellular process | 221 | 10.35129 | 0.009291 | 13.50642908 |
| establishment of localization in cell | 121 | 5.667447 | 0.009405 | 13.66104468 |
| positive regulation of cellular metabolic process | 124 | 5.807963 | 0.011023 | 15.82659225 |
| spliceosomal snRNP biogenesis | 9 | 0.421546 | 0.011208 | 16.07100539 |
| cytoskeleton organization | 67 | 3.138173 | 0.011273 | 16.15687718 |
| cell cycle phase | 64 | 2.997658 | 0.011725 | 16.75130949 |
| organelle fission | 39 | 1.826698 | 0.012372 | 17.594604 |
| melanin metabolic process | 5 | 0.234192 | 0.01356 | 19.12148803 |
| negative regulation of biological process | 237 | 11.1007 | 0.015677 | 21.77812292 |
| positive regulation of biological process | 263 | 12.3185 | 0.018318 | 24.97705087 |
| intracellular transport | 94 | 4.40281 | 0.018431 | 25.11118787 |
| protein localization | 122 | 5.714286 | 0.02007 | 27.0321731 |
| negative regulation of biosynthetic process | 83 | 3.887588 | 0.020218 | 27.20377898 |
| receptor metabolic process | 9 | 0.421546 | 0.020868 | 27.9501081 |
| positive regulation of cellular process | 240 | 11.24122 | 0.020932 | 28.0239962 |
| cell projection morphogenesis | 40 | 1.873536 | 0.021459 | 28.62346702 |
| regulation of cell death | 113 | 5.29274 | 0.023496 | 30.89845067 |
| Golgi vesicle transport | 24 | 1.124122 | 0.024763 | 32.2791679 |
| negative regulation of nitrogen compound metabolic process | 75 | 3.512881 | 0.028191 | 35.88657597 |
| ribosomal small subunit biogenesis | 5 | 0.234192 | 0.029444 | 37.15872305 |
| cellular macromolecular complex subunit organization | 54 | 2.529274 | 0.029905 | 37.62151611 |
| vesicle-mediated transport | 82 | 3.840749 | 0.030285 | 38.00024545 |
| macromolecular complex assembly | 93 | 4.355972 | 0.032677 | 40.33566812 |
| adult behavior | 17 | 0.796253 | 0.034336 | 41.90632942 |
| cell part morphogenesis | 40 | 1.873536 | 0.039669 | 46.69786024 |
| synapse organization | 13 | 0.608899 | 0.042476 | 49.068837 |
| negative regulation of organelle organization | 16 | 0.749415 | 0.04513 | 51.21946632 |
| regulation of metabolic process | 445 | 20.84309 | 0.045706 | 51.67494678 |
| microtubule cytoskeleton organization | 25 | 1.17096 | 0.047547 | 53.10387782 |
| regulation of cellular metabolic process | 426 | 19.95316 | 0.048162 | 53.57246291 |
| positive regulation of intracellular transport | 8 | 0.374707 | 0.0495 | 54.57660268 |
| cellular carbohydrate metabolic process | 56 | 2.622951 | 0.056306 | 59.37781715 |
| regulation of macromolecule metabolic process | 401 | 18.7822 | 0.057821 | 60.37973379 |
| regulation of ubiquitin-protein ligase activity during mitotic cell cycle | 14 | 0.655738 | 0.058978 | 61.12919189 |
| electron transport chain | 20 | 0.936768 | 0.060632 | 62.17772942 |
| response to protein stimulus | 19 | 0.88993 | 0.061737 | 62.86342227 |
| ribosome biogenesis | 21 | 0.983607 | 0.06376 | 64.0890217 |
| response to organic substance | 97 | 4.543326 | 0.066133 | 65.47768599 |
| transport | 325 | 15.22248 | 0.070389 | 67.84411629 |
| regulation of biosynthetic process | 365 | 17.09602 | 0.070458 | 67.88106497 |
| protein complex assembly | 70 | 3.278689 | 0.0706 | 67.95761003 |
| pigment biosynthetic process | 9 | 0.421546 | 0.071984 | 68.69110168 |
| secretion | 44 | 2.06089 | 0.073341 | 69.39535264 |
| positive regulation of epidermis development | 4 | 0.187354 | 0.074711 | 70.09108717 |
| protein metabolic process | 346 | 16.20609 | 0.079064 | 72.20520544 |
| angiogenesis | 24 | 1.124122 | 0.080839 | 73.02645312 |
| regulation of organelle organization | 33 | 1.545667 | 0.08223 | 73.65400574 |
| neuron projection development | 38 | 1.779859 | 0.082975 | 73.98440869 |
| RNA transport | 17 | 0.796253 | 0.086258 | 75.39487976 |
| establishment of RNA localization | 17 | 0.796253 | 0.086258 | 75.39487976 |
| negative regulation of protein complex disassembly | 9 | 0.421546 | 0.091401 | 77.46194525 |
| carbohydrate metabolic process | 72 | 3.372365 | 0.091823 | 77.62415699 |
| ribonucleoprotein complex assembly | 13 | 0.608899 | 0.092706 | 77.95991301 |
| nucleobase, nucleoside, nucleotide and nucleic acid transport | 19 | 0.88993 | 0.093922 | 78.41450659 |
| establishment of protein localization | 101 | 4.730679 | 0.098244 | 79.96060997 |
| positive regulation of cell death | 60 | 2.810304 | 0.098438 | 80.02775583 |
| vesicle targeting | 6 | 0.28103 | 0.099625 | 80.43234546 |

All categories identified are shown.
