# Supplementary material for: Deficiency in Nucleotide Excision Repair Family Gene Activity, Especially ERCC3, Is Associated with Non-Pigmented Hair Fiber Growth
Source: PLoS One. 2012 May 16;7(5):e34185. doi: 10.1371/journal.pone.0034185 (PMC3353974; doi:10.1371/journal.pone.0034185)
Supplement: Table S4 — Differences in Gene Ontology categorization analysis between non-pigmented hair sheath versus pigmented hair sheath at GO level 3. All categories identified are shown. (DOC) [file pone.0034185.s004.doc]

**Supporting Information**

***Table S4****.* ***Differences in Gene Ontology categorization analysis between non-pigmented hair sheath versus pigmented hair sheath at GO level 3***

| **GO category** | **Number of genes in Category** | **Percentage of total genes in gene set** | **P-Value** | **FDR** |
| --- | --- | --- | --- | --- |
| regulation of cell communication | 45 | 8.840864 | 0.002162 | 3.096948 |
| response to radiation | 13 | 2.554028 | 0.008608 | 11.80725 |
| actin polymerization or depolymerization | 4 | 0.785855 | 0.013974 | 18.49693 |
| positive regulation of cell communication | 17 | 3.339882 | 0.018206 | 23.43554 |
| regulation of cellular response to stress | 8 | 1.571709 | 0.022168 | 27.80499 |
| negative regulation of protein complex disassembly | 5 | 0.982318 | 0.024668 | 30.44239 |
| response to virus | 8 | 1.571709 | 0.029099 | 34.8962 |
| defense response | 26 | 5.108055 | 0.029468 | 35.25463 |
| detection of abiotic stimulus | 6 | 1.178782 | 0.031586 | 37.27844 |
| secretion | 15 | 2.946955 | 0.035197 | 40.59325 |
| negative regulation of cell communication | 13 | 2.554028 | 0.038874 | 43.79992 |
| amine transport | 8 | 1.571709 | 0.042024 | 46.41839 |
| regulation of protein complex disassembly | 5 | 0.982318 | 0.0494 | 52.1115 |
| detection of external stimulus | 6 | 1.178782 | 0.050724 | 53.07195 |
| cell cycle arrest | 7 | 1.375246 | 0.061746 | 60.39783 |
| negative regulation of immune effector process | 3 | 0.589391 | 0.068825 | 64.52555 |
| regulation of response to stress | 13 | 2.554028 | 0.071373 | 65.91075 |
| excretion | 5 | 0.982318 | 0.072564 | 66.54037 |
| signal transduction | 90 | 17.68173 | 0.072906 | 66.71924 |
| cell-cell adhesion | 13 | 2.554028 | 0.07427 | 67.42417 |
| carbohydrate catabolic process | 7 | 1.375246 | 0.076677 | 68.63351 |
| regulation of response to stimulus | 19 | 3.732809 | 0.08434 | 72.21173 |
| positive regulation of protein secretion | 4 | 0.785855 | 0.099743 | 78.28373 |

All categories identified are shown.
