# Supplementary material for: Deficiency in Nucleotide Excision Repair Family Gene Activity, Especially ERCC3, Is Associated with Non-Pigmented Hair Fiber Growth
Source: PLoS One. 2012 May 16;7(5):e34185. doi: 10.1371/journal.pone.0034185 (PMC3353974; doi:10.1371/journal.pone.0034185)
Supplement: Table S5 — Primer sequences for defined genes. (DOC) [file pone.0034185.s005.doc]

**Supporting Informations S5**

***Table S5. Primer sequences for defined genes***

| **Gene** | **Genbank**  **Accession No.** | | **Forward Primer** | **Reverse Primer** | **Amplicon Size** |
| --- | --- | --- | --- | --- | --- |
| ERCC3 | NM_000122 | | ATGGGCAAAAGAGACCGAGC | CTTTGGTGCCTGACTCATCCA | 157 |
| ERCC5 | NM_000123 | | TTTCACAGTACCAACTCAAAGGC | CCTTCGGATGTGTCCTGAATG | 107 |
| ERCC6 | | NM_000124 | CCACTCAAGTCAAACTCAGGAG | ATCTGATGTCGGTCGATGTGC | 219 |
| XPA | NM_000380 | | CCAGGACCTGTTATGGAATTTGA | GCTTCTTGACTACCCCAAACTTC | 317 |
| ERCC2 | NM_000400 | | TCGAGCCCTTTGACGACAGA | TGATGATGACAGACTGGAAACG | 117 |
| ERCC1 | NM_001983 | | GGGTGACTGAATGTCTGACCA | GGGTACTTTCAAGAAGGGCTC | 191 |
| ERCC4 | NM_005236 | | GGAACTGCTCGACACTGACG | GCGAGGGAGGTGTTCAACTC | 187 |
| NTPBP | NM_007266 | | CACCCAGTGTGTCTGTTGGT | GAGGTCACTATGCCGCCAT | 230 |
| HCNP | NM_020196 | | AGGAAATCATGCGGAACCAAT | TGCCCGCTCGTATAGCTGA | 104 |
| DDB2 | NM_000107 | | ACCTCCGAGATTGTATTACGCC | TCACATCTTCTGCTAGGACCG | 113 |
| POLH | NM_006502 | | GCTACTGGACAGGATCGAGTG | AACTGCACAAGGTTTATTCCTCA | 105 |
| TYR | NM_000372 | | CTGGAAGGATTTGCTAGTCCAC | CCTGTACCTGGGACATTGTTC | 106 |
| TYRP1 | NM_000550 | | TCTCTGGGCTGTATCTTCTTCC | GGCAACACATACCACTTCTCAA | 106 |
| TYRP2 | NM_001922 | | GACCTGCATTTGTTACCTGGCTAGG | CCAGTGGCAAAGTTCCAGTAG | 112 |
